# Supplementary material for: Estrogen Enhances FDFT1 Expression in Theca Cells of Chicken Hierarchical Ovarian Follicles by Increasing LSD1Ser54p Level Through GSK3β Phosphorylation at 216th Tyrosine
Source: Biomolecules. 2024 Oct 22;14(11):1343. doi: 10.3390/biom14111343 (PMC11591973; doi:10.3390/biom14111343)
Supplement: Supplementary file 1 [file biomolecules-14-01343-s001.zip › WB original images.pdf]

All protein original gels are shown in the following images. Each experiment contains three replicates. The strips used in the figures are marked with red boxes

Figure 1A

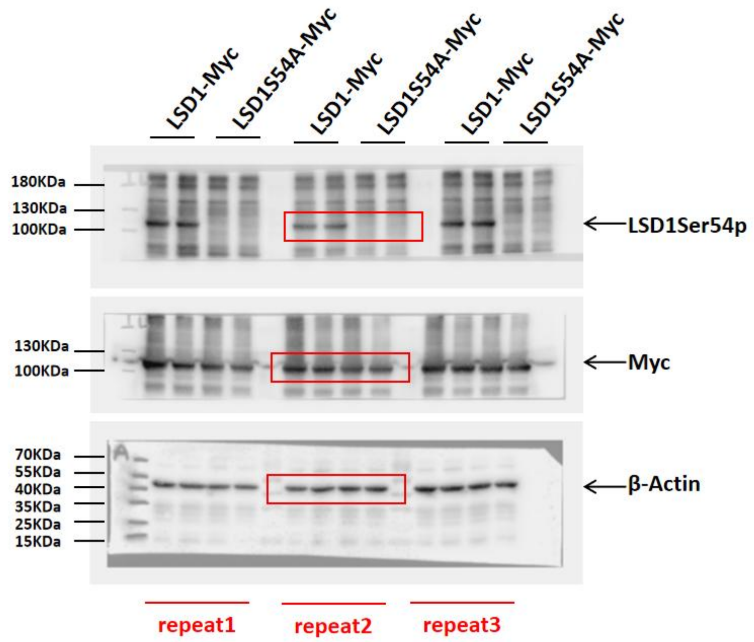

Figure 1B

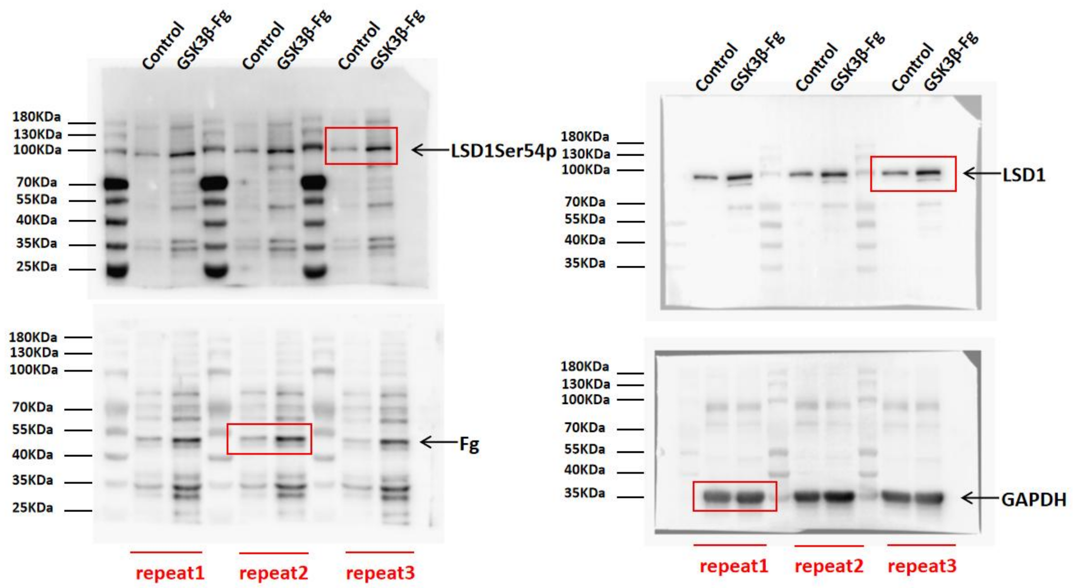

Figure 1C

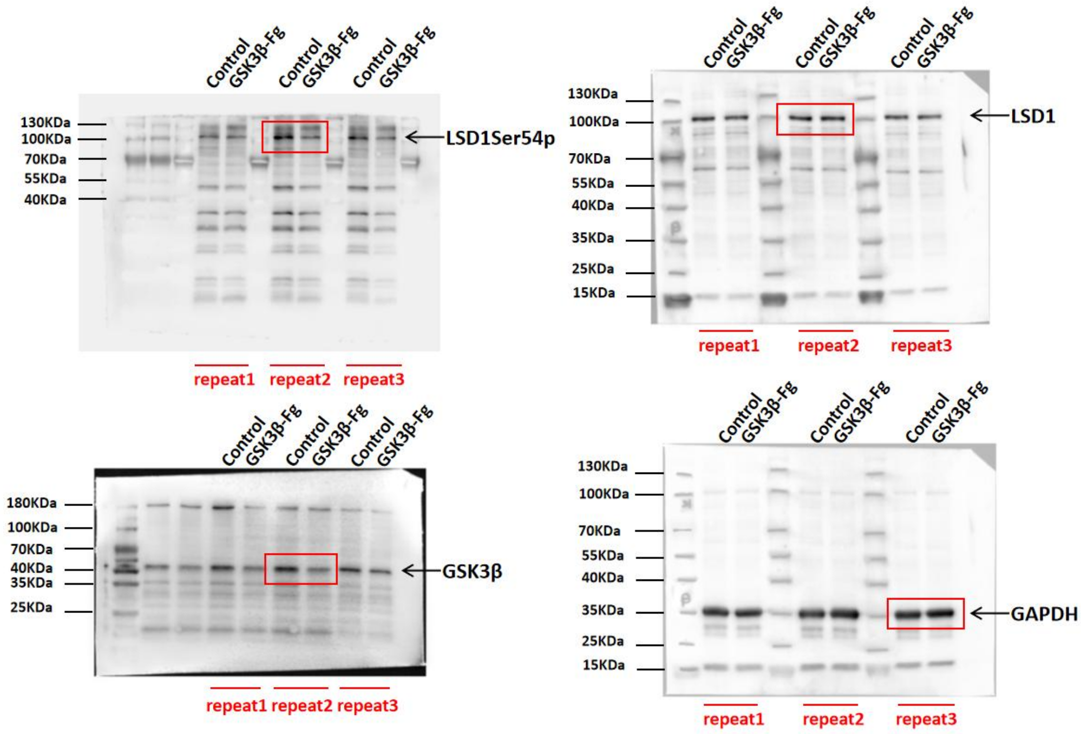

Figure 2A

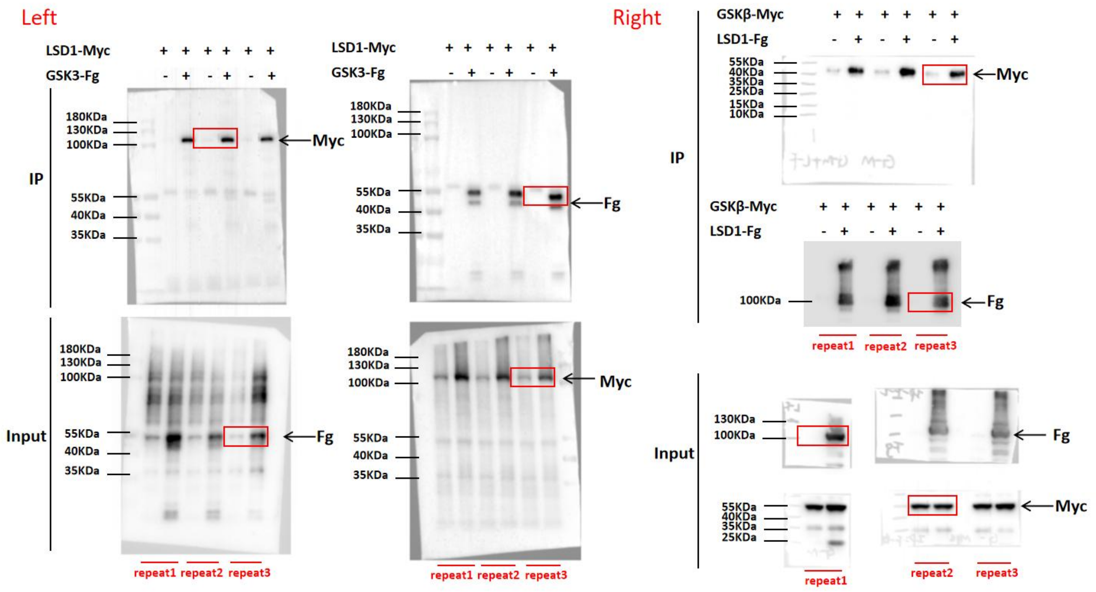

Figure 2B

Left

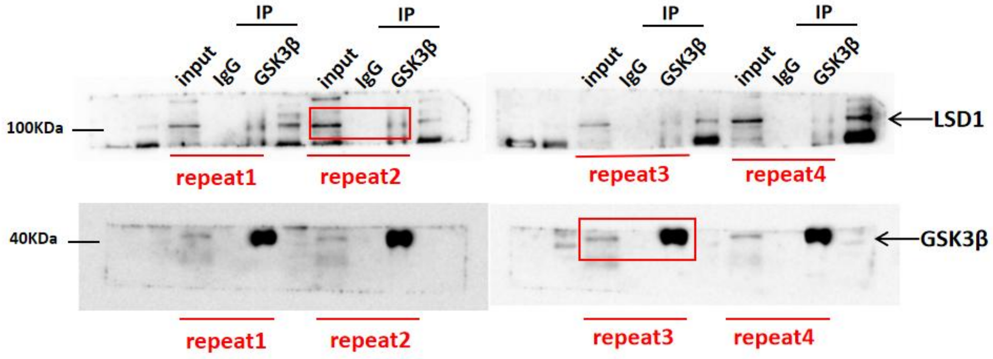

Right

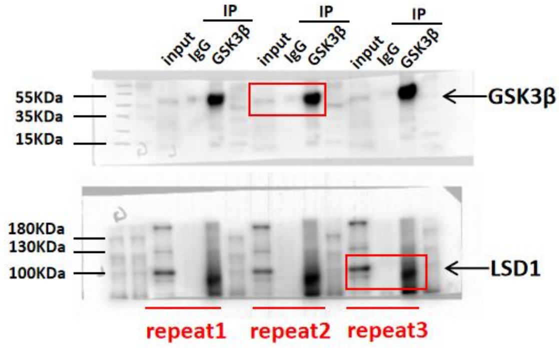

Figure 2C

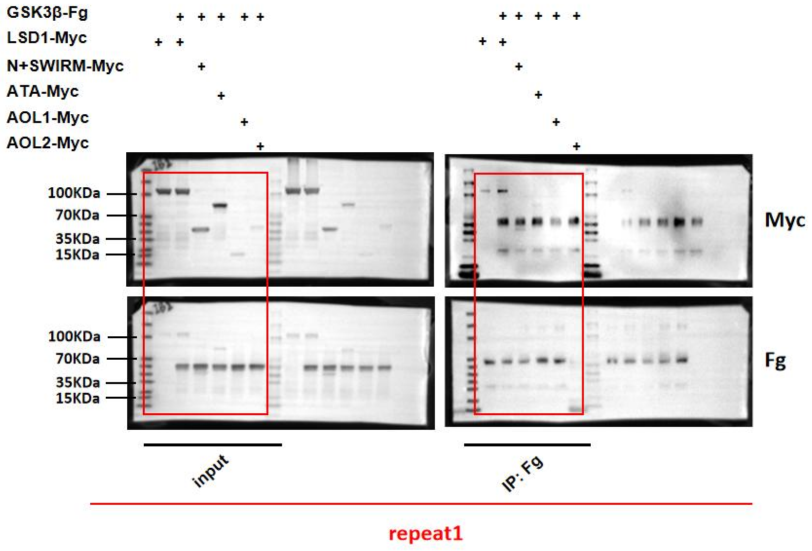

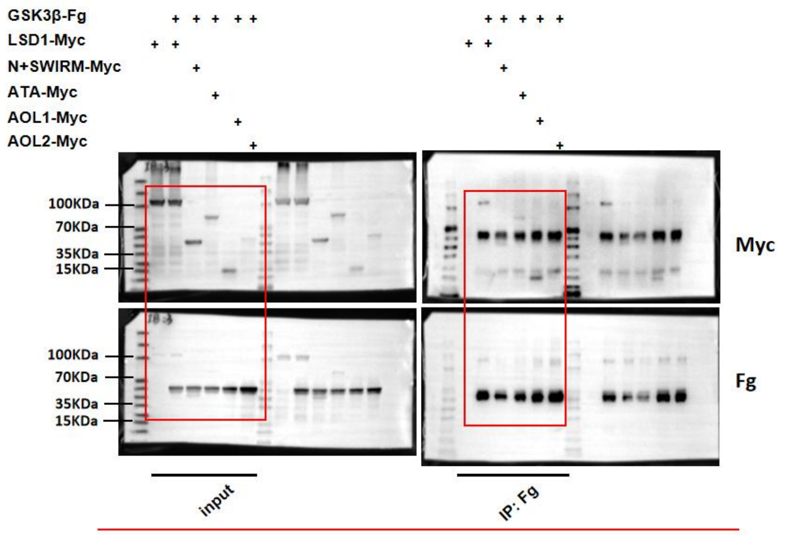

repeat2

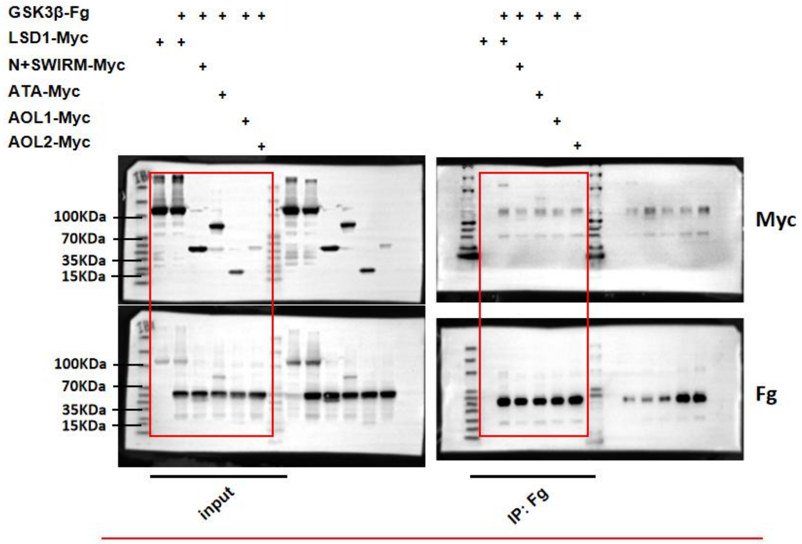

repeat3

Figure 3A

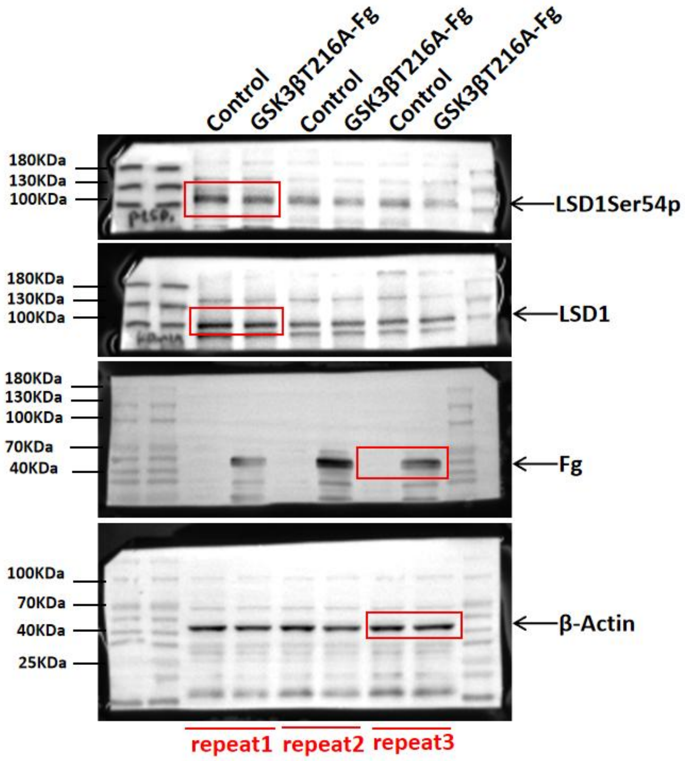

Figure 3B

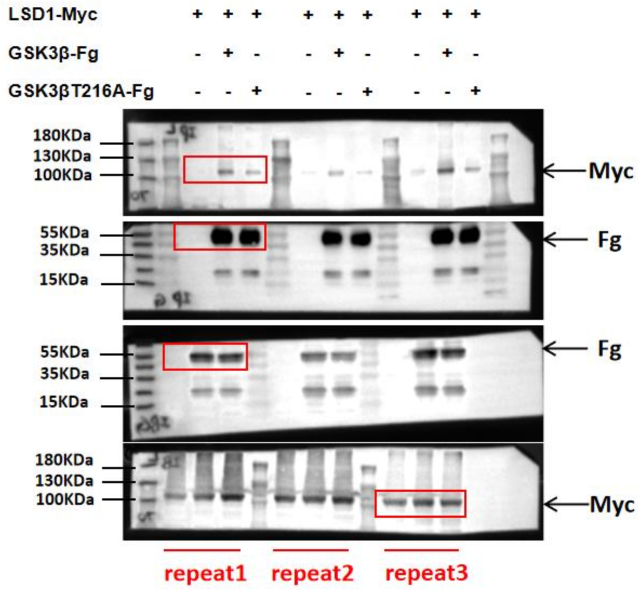

Figure 3C

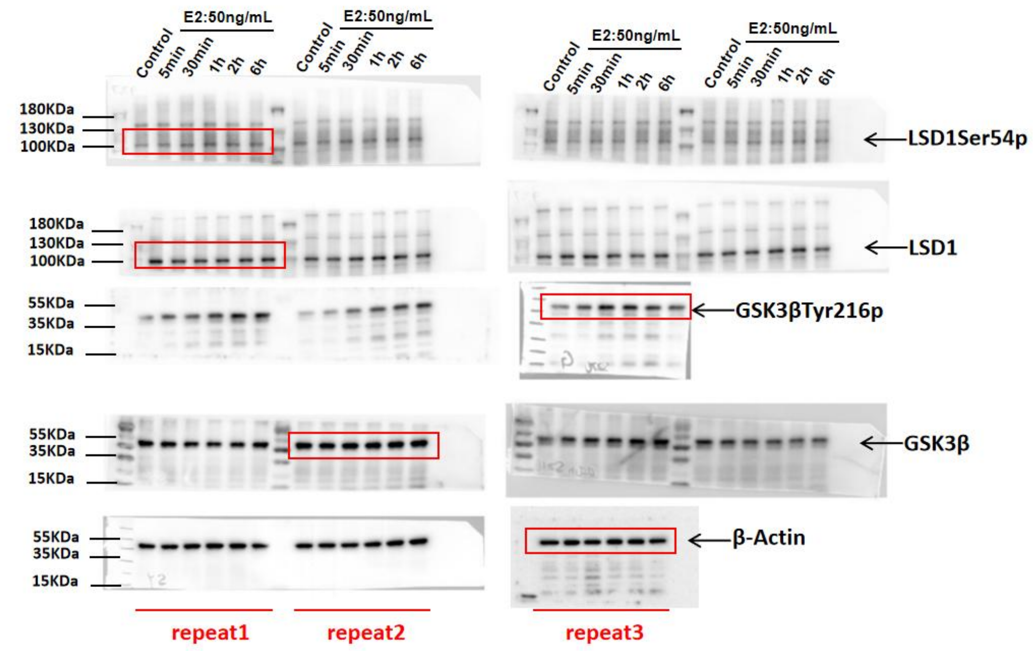

Figure 3E

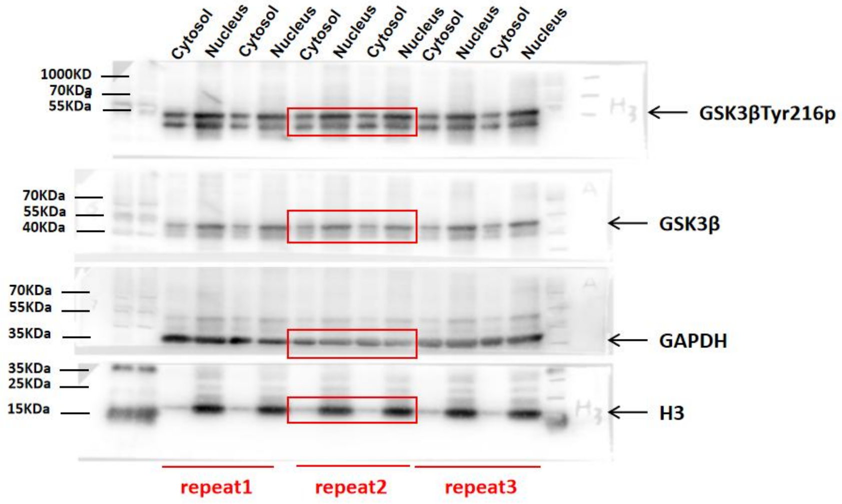

Figure 6B

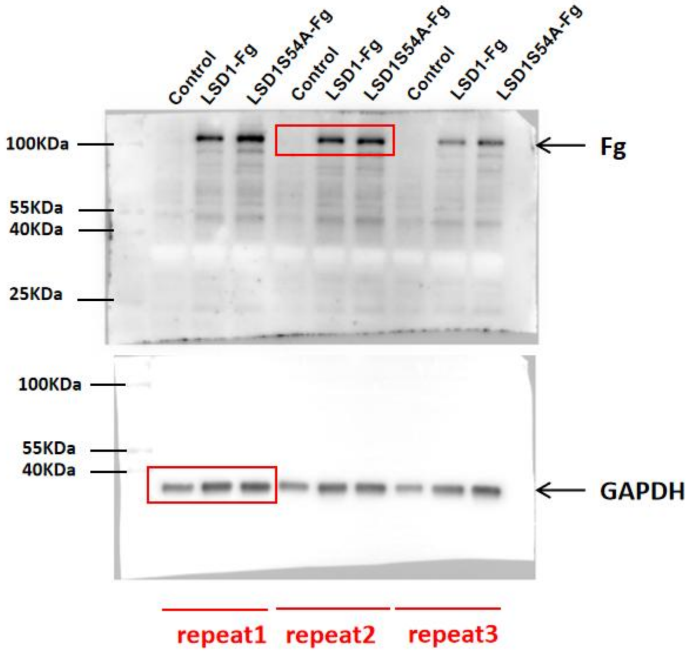

Control
LSD1-Fg
LSD1S54A-Fg
Control
LSD1-Fg
LSD1S54A-Fg
Control
LSD1-Fg
LSD1S54A-Fg
100KDa
55KDa
40KDa
25KDa
Fg
100KDa
55KDa
40KDa
GAPDH
repeat1
repeat2
repeat3
