# Supplementary material for: Estrogen Enhances FDFT1 Expression in Theca Cells of Chicken Hierarchical Ovarian Follicles by Increasing LSD1Ser54p Level Through GSK3β Phosphorylation at 216th Tyrosine
Source: Biomolecules. 2024 Oct 22;14(11):1343. doi: 10.3390/biom14111343 (PMC11591973; doi:10.3390/biom14111343)
Supplement: Supplementary file 1 [file biomolecules-14-01343-s001.zip › Supplementary Data S1.pdf]

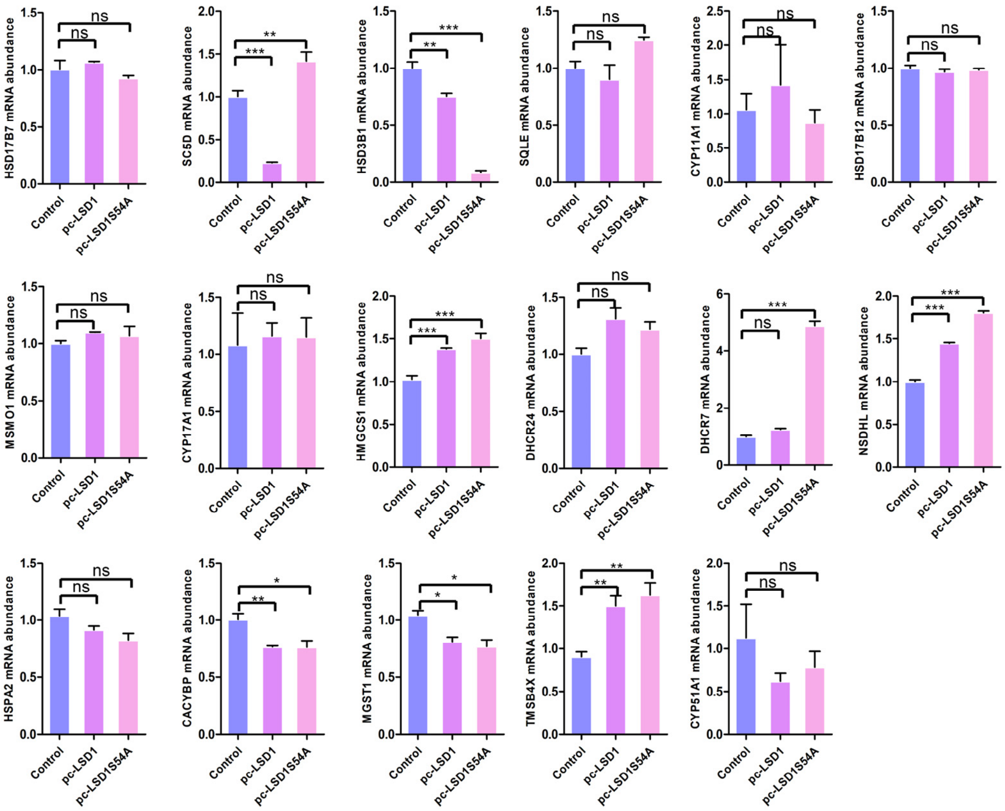

Fig. S1. Analyze the LSD1Ser54p target gene regulated by estrogen through RNA-seq and CUT&RUN sequencing results. The wild-type LSD1 and LSD1S54A mutants were overexpressed in Post-TC, qPCR was used to detect their expression changes, namely *HSD17β7*, *SC5D*, *HSD3β1*, *SQLE*, *CYP1A1*, *HSD17β12*, *DHCR7*, *MSMO1*, *CYP17A1*, *HMGCS1*, *DHCR24*, *NSDHL*, *CYP51A1*, *HSPA2*, *CACYBP*, *MGST1*, *TMSB4X*. $^{*}P < 0.05$, $^{**}P < 0.01$, $^{***}P < 0.001$, $^{ns}P > 0.05$.
