# Supplementary material for: Estrogen Enhances FDFT1 Expression in Theca Cells of Chicken Hierarchical Ovarian Follicles by Increasing LSD1Ser54p Level Through GSK3β Phosphorylation at 216th Tyrosine
Source: Biomolecules. 2024 Oct 22;14(11):1343. doi: 10.3390/biom14111343 (PMC11591973; doi:10.3390/biom14111343)
Supplement: Supplementary file 1 [file biomolecules-14-01343-s001.zip › Supplementary Data S2.pdf]

Name	Abbreviation	Function
Estrogen	E2	A type of steroid related hormone that promotes the formation and development of primordial follicles
glycogen synthase kinase 3 beta	GSK3β	A type of kinase that is widely involved in intracellular signal transduction
lysine demethylase 1A	LSD1	Histone demethylase,by removing the methyl and dimethyl groups of histones H3K4 and H3K9, it functions effectively
farnesyl-diphosphate farnesyltransferase 1	FDFT1	The rate limiting enzyme in cholesterol biosynthesis process

Data S2. The signaling molecules, proteins and genes involved in this study.
